# Screening and identification of potential PTP1B allosteric inhibitors using *in silico* and *in vitro* approaches

**DOI:** 10.1371/journal.pone.0199020

**Published:** 2018-06-18

**Authors:** Ranajit Nivrutti Shinde, G. Siva Kumar, Shahbaz Eqbal, M. Elizabeth Sobhia

**Affiliations:** Department of Pharmacoinformatics, National Institute of Pharmaceutical Education and Research, S.A.S. Nagar, Punjab, India; Wake Forest University, UNITED STATES

## Abstract

Protein tyrosine phosphatase 1B (PTP1B) is a validated therapeutic target for Type 2 diabetes due to its specific role as a negative regulator of insulin signaling pathways. Discovery of active site directed PTP1B inhibitors is very challenging due to highly conserved nature of the active site and multiple charge requirements of the ligands, which makes them non-selective and non-permeable. Identification of the PTP1B allosteric site has opened up new avenues for discovering potent and selective ligands for therapeutic intervention. Interactions made by potent allosteric inhibitor in the presence of PTP1B were studied using Molecular Dynamics (MD). Computationally optimized models were used to build separate pharmacophore models of PTP1B and TCPTP, respectively. Based on the nature of interactions the target residues offered, a receptor based pharmacophore was developed. The pharmacophore considering conformational flexibility of the residues was used for the development of pharmacophore hypothesis to identify potentially active inhibitors by screening large compound databases. Two pharmacophore were successively used in the virtual screening protocol to identify potential selective and permeable inhibitors of PTP1B. Allosteric inhibition mechanism of these molecules was established using molecular docking and MD methods. The geometrical criteria values confirmed their ability to stabilize PTP1B in an open conformation. 23 molecules that were identified as potential inhibitors were screened for PTP1B inhibitory activity. After screening, 10 molecules which have good permeability values were identified as potential inhibitors of PTP1B. This study confirms that selective and permeable inhibitors can be identified by targeting allosteric site of PTP1B.

## Introduction

Protein tyrosine phosphatases (PTPs) are a group of 107 enzymes that dephosphorylate phosphotyrosine residues of the protein substrates.[1,2] These enzymes are the key regulatory components in cellular functions and signal transduction pathways.[3,4] The PTP superfamily has a highly conserved active site motif C(X)5R, commonly known as PTP signature motif.[5] Protein tyrosine phosphatase 1B (PTP1B) is a representative member of this superfamily. PTP1B is a widely expressed cytosolic soluble protein with a molecular weight around 50 kD. In the native form, PTP1B consists of 435 amino-acid residues that are divided into three regions: N-terminal catalytic region or PTP domain (Residues 1–300), regulatory region (residues 300–400) and C-terminal membrane localization region (residues 400 to 435).[6,7] Activity of PTP1B is regulated by a variety of post-translational mechanisms, including phosphorylation of serine and tyrosine residues at various sites within of catalytic and transmembrane domains, oxidation of Cys215 due to reactive oxygen species, and spatial separation from its plasma membrane-localized substrates. It is involved in multiple signal transduction pathways acting as protein dephosphorylating enzyme.[8]. It has been established as a key enzyme in the negative regulation of insulin signalling pathway.[9–11]

The three-dimensional (3D) structures of the PTP domains are remarkably similar over tyrosine-specific PTPs and the dual-specificity PTPs despite the variation in their sequences and substrate specificity. The PTP domain of PTP1B is composed of a highly twisted mixed β-sheets surrounded by α-helices from both sides (S1 Fig). Regulatory region containing two proline-rich motifs spanning 301–315 and 386–397, serve as binding sites for proteins such as p130(Cas), Grb2, and Crk.[12] The structure of this region is believed to impart substrate specificity to PTP1B. C-terminal region is responsible for the binding the enzyme to the cytoplasmic face of the endoplasmic reticulum.[13] For crystallization and enzymatic assays, shorter versions of PTP1B (298 or 321 residues) are usually employed. Majority of PTP1B crystal structures solved have 298 amino acids and full length (1–435) structure is yet to be solved. The 298 residue version is composed of a single domain, organized in eight α helices and eleven β strands (S1 Fig).[6] R loop (Val113–Ser118), lysine loop (Leu119–Cys121), WPD loop (Thr177–Pro185), S loop (Ser201–Gly209), Q loop (Ile261–Gln262), α3 helix (Glu186–Glu200), α6 helix (Ala264–Ile281) and α7 helix (Val287–Ser295) play a critical role in dephosphorylation of phosphotyrosine.[14–18] PTP1B dephosphorylates the phosphotyrosine of IR activation loop as well as several docking proteins and serves as a negative regulator of the tyrosine phosphorylation cascade integral to the insulin signaling pathway.[19,20] The *in vivo* effects observed on the loss of PTP1B are specific to the components of the insulin action.[9,10] These studies have placed PTP1B as potential target for diabetes, which is a metabolic disorder. Various PTP homologs are used to check the selectivity of the PTP1B inhibitors. Among the close homologues of PTP1B, TCPTP has been of special interest in selectivity studies because it is the most homologous phosphatase to PTP1B with 72% identity in the catalytic domain (TCPTP residues 43 to 288). Along with sequence similarity, it is structurally very similar to the PTP1B (< 0.5 Å catalytic domain residue backbone RMSD).[21] As TCPTP inhibition is related to bone marrow destruction, B cell lymphopoiesis, erythropoiesis, as well as impaired T and B cell functions, selective inhibition of PTP1B is highly desirable.[22]

PTP1B catalyzes dephosphorylation in two stages.[23,24] In the first stage, catalytically competent enzyme-substrate complex is formed between the enzyme and phosphotyrosine containing substrate. The enzyme undergoes the conformational change during the binding of substrates. It moves the WPD loop from open conformation to closed conformation, optimizing the interactions of its residues, Phe180 and Asp181, with phosphotyrosine. The nucleophile Cys215, underneath the phosphoryl group, makes the nucleophilic attack on the phosphorus atom and binds covalently to phosphate moiety. The Asp181 then breaks the bond between the phenyl and the phosphate groups by acting as a general acid, resulting in the protonation of phenolic oxygen of the product. In the second step, the bond between the enzyme and the phosphate group is broken by hydrolysis reaction. The deprotonated Asp181 acts as a general base, extracting proton from a water molecule. The activated water molecule then makes nucleophilic attack on the phosphorus atoms of phospho-enzyme complex. It sets free inorganic phosphate from the enzyme, leading to completion of catalytic process and opening of WPD loop.

In 2004, Wiesmann et al. revealed novel binding site of PTP1B, located at a distance of ~20 Å from the active site, through co-crystallization of three non-competitive inhibitors (PDB ID: 1T48, 1T49, 1T4J bound to Inhibitor-1, Inhibitor-2 and Inhibitor-3, respectively).[25] These inhibitors are shown in S2 Fig. The site is an adaptive binding site formed in the presence of suitable inhibitor through conformational rearrangements. Inhibitors are believed to displace the Trp291 of α7 helix and occupy its position. Two other helices, α3 and α6 boarder this site and their residues Leu192, Asn193, Phe196, Glu276, Phe280 and Trp291 interact with the inhibitors. All members of PTP family share such a high degree of structural conservation in the phosphotyrosine binding pocket, designing selective and permeable inhibitor for PTP1B poses a great challenge. These hurdles can be easily overcome by designing the molecules such that they bind to the allosteric site. PTP1B allosteric site is not conserved and comprises of more hydrophobic residues as compared to the active site. Though this site is present in TCPTP, it has few differing residues compared to PTP1B viz. Cys278, Ile285, and Arg288 in place of Phe280, Val287, and Gln290, respectively.[26,27] Thus, the allosteric site in PTP1B present a benchmark strategy for developing inhibitors with improved cell membrane permeability. In addition, targeting non-conserved residues can afford an opportunity to circumvent selectivity problems associated with the inhibitors binding at the active site.

Ligand that binds at the allosteric site inhibits PTP1B with an allosteric mechanism. This mechanism is described as follows; during the catalysis, WPD-loop shows maximum movement whereby it travels a distance of several angstrom from its initial position (“open” state) to bend over phosphotyrosine (“closed” state). Bending of the loop bring the residues essential for catalysis into close proximity with the phosphotyrosine. Mechanistically, allosteric inhibitor locks the PTP1B in ‘open’ conformation and thereby prevents it from converting into a ‘close’ conformation which is essential for substrate catalysis. It has shown that formation the hydrogen bond network within the helices of α3-α6-α7 is essential in the closure of WPD loop.[28] However, allosteric inhibitor gets accommodated into these helices destabilizing the network of hydrogen bonds.[29] This is one of the mechanism by which inhibitor forbid the closure of WPD loop.

Since the discovery of allosteric site, several studies including molecular dynamics, pharmacophore development, helix modelling, and energy analysis have been performed on the allosteric site and allosteric inhibitors. A study by Kamerlin et al. explored the flexibility of the WPD loop in presence and absence of an allosteric inhibitor.[15] Another study by the same group observed the changes in the various regions of PTP1B by simulating the movement of WPD loop from open to close and vice versa.[16] Bharatham et al. group studied the interaction patterns between Inhibitor-2 and allosteric site residues of PTP1B.[30] They used α7 helix flexibility associated with adaptive binding to generate dynamic pharmacophores, which were subsequently used to retrieve potential allosteric inhibitors. Alakent et al. explored the role of α7 helix, a core region of allosteric site, through accessing the mobility of the various regions in the presence and absence of the helix using MD.[31] It was concluded that the helix is critical for the conformation and dynamics of WPD loop. Another study investigated the molecular mechanism of two non-competitive PTP1B inhibitors, chlorogenic acid and cichoric acid. Dynamic interactions between the inhibitors and allosteric site and restricted mobility of WPD loop helped them to conclude that the inhibitors exhibit PTP1B inhibition through potent binding at the allosteric site.[32] In a similar work, Lee et al. used docking method to prove the amentoflavone as an allosteric inhibitor of PTP1B.[33] In a recent allosteric inhibitor binding free energy analysis study by Cui et al. revealed that the van der Waals contribution exhibits stronger binding affinity and hinders the WPD loop movement.[34]

α6-α7 loop and α7 helix are the missing or discorded secondary structure elements in the co-crystal structures of PTP1B with Inhibitor-1, 2 and 3 and thus lack the information about the interaction features accessible for the inhibitors. A MD study by Bharatham et al. has observed consistent H-bond interactions between Inhibitor-2 and Ser295 of α7 helix, pointing it as an essential H-bond donor feature.[30] Same study also has shown Trp291 as a part of hydrophobic cavity that accommodates the benzofuran ring of inhibitors revealing it as hydrophobic feature. Per residue contribution energy analysis carried out by Cui et al. has proposed residues Trp281, Lys292 and Glu293 as most important residues as they provide a stronger interactions for the binding of Inhibitor-1.[34] Additionally, Val287, Gln288 were observed to have weaker interactions with the same Inhibitor. Interactions of the most potent allosteric inhibitor, Inhibitor-3, with the missing helix and loop residues is not studied yet. Such study can provide more insightful information for the rational design of novel inhibitor. In this regard, we have performed MD studies three allosteric inhibitors in presence PTP1B as well as TCPTP. These studies have shown residues viz., Val287, Trp291 and Gln288 of α7 helix and Gly283, Asp284, Ser285, and Ser286 of α6-α7 loop as essential residues in the binding of PTP1B inhibitors. Similarly Ile285, Trp289, Gln286 and Lys290 residues of α7 helix in TCPTP interact with Inhibitor-1, 2 and 3. Nature of interactions these residues offer can be understood by use of receptor based pharmacophore study. In addition they pharmacophore considers conformational flexibility of the residues. Subsequently pharmacophore hypothesis can be used for the identification of potentially active inhibitors by virtually screening the large compound databases. Hence, we aimed to find the pharmacophore features essential for ligands to act as allosteric inhibitors of PTP1B. Additionally, considering the adaptive nature of allosteric site, we used conformations generated during molecular dynamics simulations of Inhibitor-1, 2 and 3 complexes to develop a dynamic pharmacophore for PTP1B and TCPTP inhibitors.

## Materials and methods

### Modelling of allosteric site of PTP1B and TCPTP

Apo form of PTP1B structure has α6-α7 loop (282–286), α7 helix (287–295) connected to it and 296–298 as terminal residues. However in presence of allosteric inhibitors (S2 Fig), residues of this helix and loop were either disordered (PDB ID: 1T48) or absent (PDB ID: 1T49 and 1T4J).[25] Therefore, these residues were modelled in each of the co-crystal structures i.e. 1T48, 1T49 and 1T4J using the procedure as described in Shinde et al. [27] The resulted complexes were named as Model-1 (PTP1B—Inhibitor-1 complex), Model-2 (PTP1B—Inhibitor-2 complex) and Model-3 (PTP1B—Inhibitor-3 complex). Briefly, Human PTP1B structure 2F6F was used to model residues 282–292.[28] Bioactive conformations of inhibitors were constrained and residues were built around them using Modeller 9v8 program.[35] The α7 helix and α6-α7 loop was also missing in the TCPTP crystal structure 1L8K.[21] The residues 278–293 corresponding to α6-α7 loop and α7 helix were then modelled to this structure using the same PTP1B structure 2F6F.[28] Conformations of Inhibitor-1, Inhibitor-2 and Inhibitor-3 were modeled into the allosteric site by transferring their coordinates from the co-crystal structures 1T48, 1T49 and 1T4J respectively.[25] Then sequence alignment file created by MALIGN program of Modeller was edited to involve desired part of templates i.e. residues numbered 1–277 form 1L8K, residues numbered 278–293 form 2F6F and the conformation of Inhibitor-1 from 1T48 (S3 Fig). Finally structures 1T48, 2F6F, and 1L8K aligned by SALIGN program were used to build residues numbered 278–293 around Inhibitor-1. It resulted in the building of TCPTP—Inhibitor-1 complex of 293 residues (Model-4). Procedure was repeated by replacing 1T48 by 1T49 to build TCPTP—Inhibitor-2 complex (Model-5) and by 1T4J to build TCPTP—Inhibitor-3 complex (Model-6). These 6 models (Model-1 to Model-6) were then subjected to MD simulations as described in Shinde et al.[27] The trajectories then used to perfume cluster analysis.

### Cluster analysis

In the last few years, about 10 molecules were reported as PTP1B allosteric inhibitors.[25,32–34,36] Out of these, 3 PTP1B allosteric inhibitors (Inhibitor-1, Inhibitor-2 and Inhibitor-3), for which co-crystal structures are available and MD studies have been carried out, were selected for the generation of structure based pharmacophore model. The half maximal inhibitory concentration (IC_50_) of these inhibitors was 8, 22, and 350 μM respectively.

The 20 ns production MD simulations of three PTP1B complexes (Model-1, Model-2 and Model-3) and three TCPTP complexes (Model-4, Model-5 and Model-6), were used for the generation of receptor based pharmacophore models. From a total of 5000 frames saved during the MD simulation, selection of a few representative snapshots which could represent the conformational flexibility of the protein was crucial. Initially these trajectories were subjected to cluster analysis with the use of g_cluster analysis present in the GROMACS distribution.^[^^37^^,^^38^^]^ This approach generates an XPM matrix with the RMSD comparisons of each conformation with all the others present in the trajectory and is subsequently used in the generation of clusters. Further, this method counts number of neighbours for each conformation using RMSD cut-off, selects a structure with largest number of neighbours, considers all the neighbours as a cluster and eliminates them from the pool of conformations.^[^^38^^]^ This procedure is repeated for the remaining structures in the pool to find the new clusters. For each of six models, ten clusters were generated using a single linkage method in which a structure is added to a cluster when its distance to any element of the cluster is less than a specified cut-off. To generate the ten clusters different cut-off distances were used. Out of ten, the clusters which contain a maximum percentage of total conformations were selected for further analysis. The structure with the smallest average distance with the other members of same cluster was taken as the representative structure of that cluster and subsequently used for the generation of pharmacophore model.

### Structure based pharmacophore models generation

A pharmacophore is the specific arrangement of chemical features responsible for the biological activity of a molecule.[39] IUPAC defines pharmacophore as an ensemble of steric and electronic features that is necessary to ensure the optimal supramolecular interactions with a specific biological target and to trigger (or block) its biological response.[40] Some of the ligand based tools are HipHop[41] HypoGen[42] GALAHAD[43] PHASE[44], while receptor based tools are LigandScout[45], Pocket V2[46], and SBP[47]. LigandScout, an automated tool for pharmacophore generation, was used to study the interactions between the inhibitor and amino acids in the allosteric site of PTP1B. A representative conformation from each cluster of Model-1, 2 and 3 was selected and receptor based features were generated using LigandScout. From these pharmacophore features a common pharmacophore model was developed. Similar procedure was followed for the TCPTP complexes. Representative complexes were subjected to the LigandScout software for the identification of receptor based features. The generated hypotheses were then used to develop a common pharmacophore model. The developed structure based pharmacophore hypotheses were exported in hypoedit format and then converted into .chm format using hypoedit tool in the discovery studio, which was then used as a 3D query for virtual screening process.

### Validation of pharmacophore models

**Fig 1:**
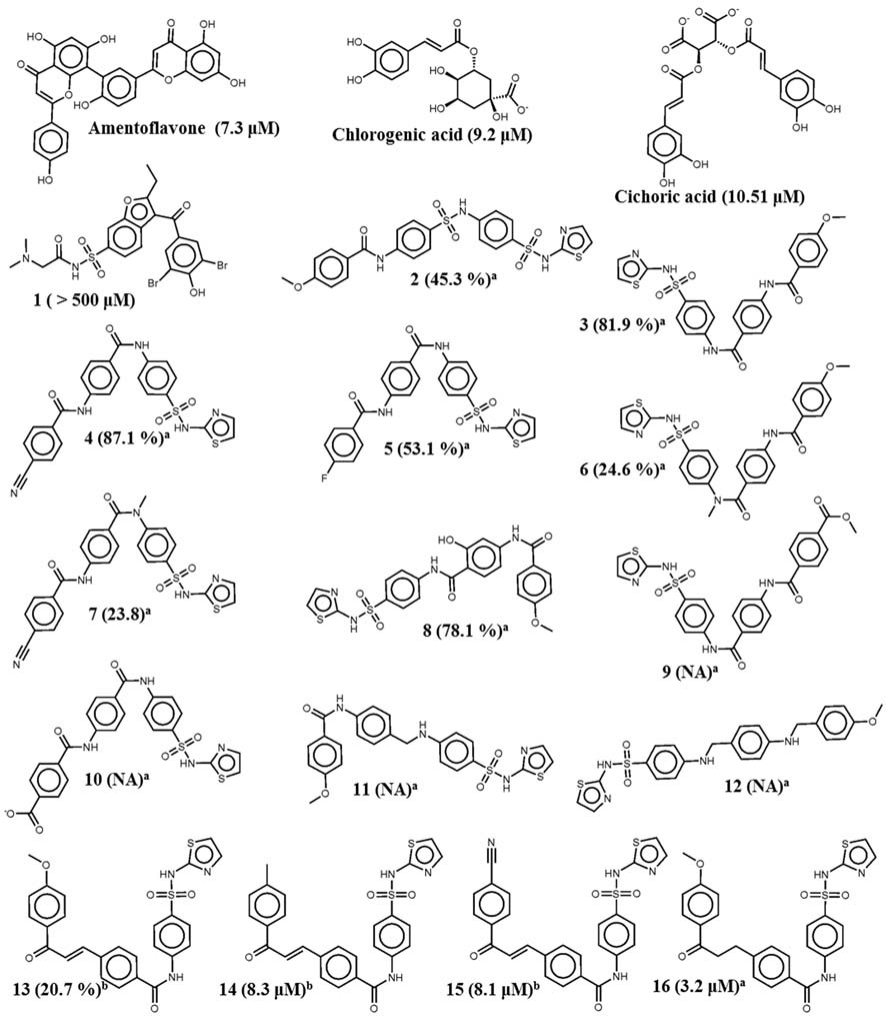
19 test molecules that are used for the validation of the pharmacophore model. [32,33,48] Values in bracket indicate activity of the molecules either in IC_50_ values or in % inhibition. ^a^ The percentage inhibition was measured under the concentration of 100 μM. ^b^ The percentage inhibition was measured under the concentration of 10 μM.

The developed pharmacophore model was validated to determine its capability in differentiating the actives from the less actives and inactives and to perform virtual screening of databases. For the validation purpose, two different methods, test set prediction and decoy test prediction were employed. In the test set predication of PTP1B, a set of 19 molecules were used (Fig 1).[32,33,48] The test molecules were classified into the active (IC_50_ < 25 μM or inhibition > 50%), less active (IC_50_ > 25 μM or inhibition < 50%) or inactive (No activity) groups based on their known inhibitory activity. The first group of active molecules consisted of Amentoflavone, Cichoric acid, Chlorogenic acid, Molecule—3, 4, 5, 8, 14, 15, and 16. The second group consisted of Molecule—1, 2, 6, 7, and 13 while the third group consisted of Molecule—9, 10, 11 and 12 (Fig 1). The molecules were sketched and minimized in Catalyst. For each molecule a maximum of 255 conformers (with an energy threshold of 4 kcal/mol) were generated and considered for model validation. Flexible method was employed for fitting the molecule to the pharmacophore hypothesis. This method ensures an exhaustive conformational mapping even for most complex molecules. Default values were used for all other parameters in the conformational analysis. All the test set molecules were mapped on the developed pharmacophore hypothesis and “FitValue” was predicted for each test set molecule. Further, for evaluation purpose, test set molecules were divided into active, less active and inactive based on the predicted FitValues. The hit rate of pharmacophore hypothesis was calculated to analyse the efficiency of developed pharmacophore hypothesis in differentiating between actives and less actives.

For further validation of the generated hypothesis, a decoy set was generated using DecoyFinder1.1.[49] Decoys are molecules that are supposed to be inactive against a target and are used to validate the performance of the virtual screening workflow. Decoys are supposed to be inactive against a target and are used to validate the performance of the virtual screening. They were selected based on the similarity of the molecules with active ligands which is calculated considering physical descriptors such as molecular weight, number of rotational bonds, hydrogen bond donor count, hydrogen bond acceptor count and the octanol–water partition coefficient. However, they do not possess any chemical descriptors representing the active ligands.[49] For each of the 12 active PTP1B inhibitors 36 decoys were generated. Total of 432 decoy molecules and 12 active inhibitors were used to calculate various statistical parameters such as accuracy, precision, sensitivity, specificity, goodness of hit score (GH), and enrichment factor (E value). Out of these, GH and E-value are the two major parameters, playing a significant role in identifying capability of the generated pharmacophore hypothesis.^[^^50^^]^

Very few molecules are known to be the allosteric inhibitors of TCPTP. Hence, only one molecule, 16, was considered in the test set for TCPTP. Molecule 16 is a PTP1B allosteric inhibitor which has shown inhibitory activity against TCPTP (IC_50_−40 μM, Fig 1).[48] Decoy test validation was not performed for the TCPTP pharmacophore because of the unavailability of the large number of active molecules.

### Virtual screening

Virtual screening of chemical databases is a fast and accurate method, which helps to identify novel and potential leads suitable for further development. Specs, database of commercially available diverse chemical molecules were used in virtual screening. The databases were first screened for their drug-likeness properties using Lipinski’s rule of five and subsequently submitted to DEREK for different toxicity filters. Fast/Flexible and Best/Flexible are the two databases searching options available in Discovery Studio. In our study, we performed virtual screening using Best/Flexible search option. The validated PTP1B pharmacophore hypothesis was used as a 3D query in database screening. The hits were selected based on the cutoff values that were obtained for the active and less active molecules used in the test set. The hits that meet this criterion were further subjected to the screening with TCPTP pharmacophore. The molecules that were not picked in the pharmacophore search and showed the FitValues less than the TPCPT active molecules were considered to be potentially PTP1B selective.

The hits obtained from pharmacophore based virtual screening were evaluated for drug-likeness properties using QED (quantitative estimate of drug-likeness) value.[51] For calculation of QED value, physicochemical properties such as MW, ALOGP, number of HBDs, number of HBAs, molecular PSA, number of ROTBs and the number of AROMs were calculated. The un-weighted and weighted QED values were calculated based on the above mentioned molecular properties by using following formulae:

$$\text{QED}=\text{exp}\left(\frac{1}{\text{n}}\sum_{\text{i}=1}^{\text{n}}\;\text{ln}\;{\text{d}}_{\text{i}}\right)$$ (1)

$${\text{QED}}_{\text{w}}=\text{exp}\left(\frac{\sum_{\text{i}=1}^{\text{n}}{\text{w}}_{\text{i}}\;\text{ln}\;{\text{d}}_{\text{i}}}{\sum_{\text{i}=1}^{\text{n}}{\text{w}}_{\text{i}}}\right)$$ (2)

Where d is the derived desirability function corresponding to different molecular properties; w is the weight applied to each function and n is the number of molecular properties.[51] Hit molecules that passed all of these tests were used in molecular docking analysis using Glide5.5

### Molecular docking

To investigate the detailed intermolecular interactions between the virtual hits and PTP1B, an automated docking program Glide5.5 was used.[52] The molecules were docked into the allosteric site of Model-3. Before using these models for docking, a grid of 10 Å was created around the allosteric inhibitors. The extra precision (XP) mode and other default parameters of Glide software were used for the docking studies. The model providing high docking score and pose similar to the 3D pharmacophore was selected for the molecular dynamics simulation. Before carrying out MD of the complexes the numbers of molecules were reduced by applying an ADMET filter.

### Molecular dynamics

Finally, the docked complex of hit molecules was subjected to molecular dynamics simulations to confirm the stability of complexes^10^. Before performing the MD simulation pTyr was modelled at the active site. Binding free energy calculations were averaged over 500 frames taken at the interval of 40 ps over the production run of 20 ns.

### Permeability analysis

Allosteric inhibitors are believed to have good permeability. Permeability was assessed by analysing the pharmacokinetic parameters HIA, caco-2 permeability using QikProp.^25^

## Results and discussion

### Cluster analysis

5000 conformations generated during the 20 ns of production simulation of PTP1B models, Model-1, 2 and 3, were clustered. Three clusters were identified as prominent clusters for Model-1; two clusters were identified as prominent clusters for Model-2 as well as for Model-3 (Table 1).

**Table 1:** Prominent clusters obtained in PTP1B models.

	Model-1			Model-2		Model-3	
	Cluster-1	Cluster-2	Cluster-3	Cluster-1	Cluster-2	Cluster-1	Cluster-2
**conformations** **out of 5000**	2645	1091	907	2803	1607	3244	771

In a similar way clustering was performed for the TCPTP models, Model-3, Model-4 and Model-5. Details of these clusters are given in Table 2.

**Table 2:** Prominent clusters obtained in TCPTP models.

	Model-4	Model-5		Model-6	
	Cluster-1	Cluster-1	Cluster-2	Cluster-1	Cluster-2
**conformations** **out of 5000**	3414	2032	1601	3450	1025

### Pharmacophore development for PTP1B inhibitors

A representative set of cluster conformations of Model-1, Model-2 and Model-3 were used for the purpose of generating of receptor based features. There were 3, 2 and 2 representative conformations for Model-1, Model-2 and Model-3 respectively. The seven pharmacophore hypotheses are shown in the S4 Fig.

The three Model-1 representatives, showed five pharmacophore features in Inhibitor-1. They correspond to the two bromine atoms, one ethyl side chain, one benzofuran ring and one carbonyl oxygen atom (S4 Fig). Among these features, first three groups, two bromine atoms and an ethyl side chain were hydrophobes; benzofuran ring was an aromatic-ring feature while, carbonyl oxygen atom was a hydrogen acceptor feature. Inhibitor-2 also showed the same features with exception of a bromine atom hydrophobe. In addition to these, it also showed four more features corresponding to the benzofuran sulfonyl nitrogen (as hydrogen bond donor), phenyl sulfonyl ring (as aromatic ring), phenyl sulfonamide nitrogen (as hydrogen bond donor), and phenyl sulfonyl oxygen atom (as hydrogen bond acceptor). Thus a total of eight pharmacophore features were identified for Inhibitor-2. In case of Model-3, three more features were noticed. The three additional features were thiophene ring (as aromatic ring), thiophene ring nitrogen (as aromatic ring) and benzofuran ring (as П-П stacking ring).

**Fig 2:**
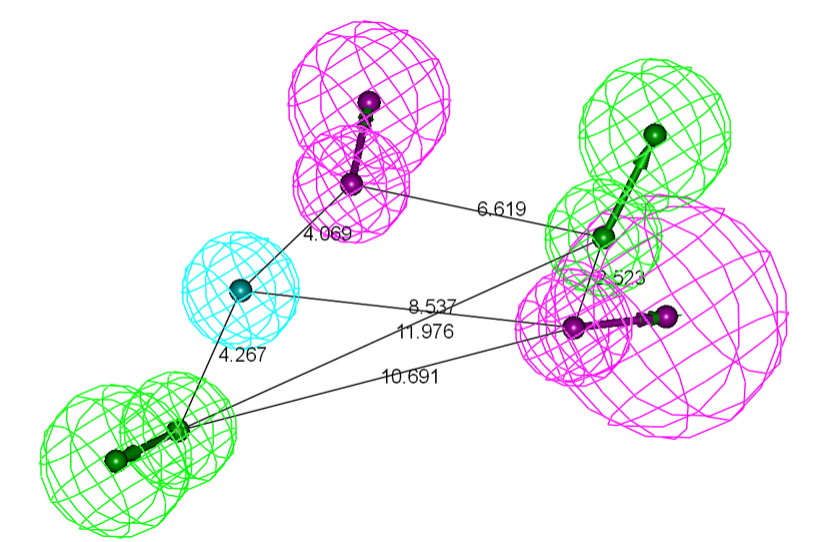
Receptor based pharmacophore model (Hypo-1) of PTP1B allosteric inhibitors generated by LigandScout. Pharmacophore features are: hydrogen-bond acceptor (green), hydrophobic (cyan), and hydrogen-bond donor (magenta).

In all hypotheses, an H-bond acceptor feature corresponding to the carbonyl oxygen of inhibitors pointed towards the Asn193 (except cluster 2 representative structure of Model-2). This interaction, between Asn193 and carbonyl oxygen of inhibitor, was stable for Inhibitor-1 and 3 during the 20 ns MD simulations performed. For inhibitor-2, this interaction was terminated just after the initiation of production phase. A study of the simultaneous binding of the three inhibitors also reported this interaction as the potential reason for the higher activity of the Inhibtor-3 over Inhibitor-1 and 2. Hence, this H-bond donor feature was considered to be essential for the activity of the inhibitors. Another H-bond donor feature pointed towards the Glu276 was common in all the hypotheses of the Model-2 and Model-3 which contain Inhibitor-2 and Inhibitor-3 respectively. The corresponding chemical features of inhibitor-2 and 3 was benzofuran sulfonamide nitrogen. Absence of this interaction in Inhibtor-1 is considered to be one of the reasons for its lower activity. Hence, this feature was also considered to be essential for the activity of the inhibitors. The third feature, which was present in all the seven hypotheses, was the hydrophobic feature that interacts with the Phe280. This residue consistently interacts with the three inhibitors contributing van der Waals energy of -3.38 kcal/mol, -6.71 kcal/mol, 7.49 kcal/mol for the binding of Inhibitor-1, 2 and 3, respectively. This feature was the third feature considered in the pharmacophore development. Another two residues that provided H- bond acceptor feature were found to be Asp284 and Val287. Asp284 and Val287 formed H- bonds with the phenyl sulfonamide oxygen of Inhibitor-2 and Inhibitor-3, respectively (Model-2 and Model-3). Overlapping of Model-2 and Model-3 hypotheses aligned these H-bond accepter features. Hence this feature was also considered in the pharmacophore development. Residue Phe280 provide H-bond accepter feature through backbone carbonyl oxygen in Model-2 hypothesis. It interacts with the phenylsulfonamide nitrogen of the Inhibitor-2. Residue Asp284 points to the H-bond donor feature in the hypothesis of Model-3. The complementary accepter feature present on the Inhibitor-3 is phenyl sulfonamide nitrogen. In the MD study carried out for Model-3, this interaction was observed to be a strong H-bond with the occupancy of 93.28%. When the Model-1 and Model-2 hypothesis were aligned, the H-bond donor features matched with each other. Hence this feature was also considered as part of a final pharmacophore. All of the seven hypotheses generated from the seven representative complexes were aligned and the above stated common features were merged to create a common hypothesis. Thus, the final receptor based pharmacophore (Hypo-1) consisted of five features namely 2 H-bond donor, 2 H-bond acceptors and one hydrophobe, as shown in Fig 2.

**Fig 3:**
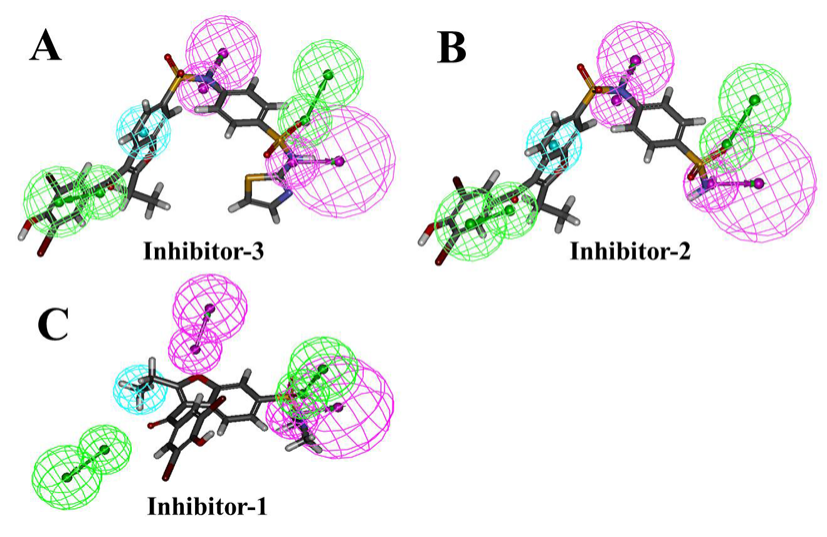
Hypo-1 mapping with the Inhbitior-3 (A) and Inhbiitor-2 (B), matching all the features. Inhibitor-1 (C) showing unmatched H-bond acceptor feature.

All features of the Hypo-1 were nicely mapped with the corresponding chemical functional groups of Inhibitor 2 and 3 (active molecules) in the training set. In contrast, Inhibitor 1 (less active molecules) mapped four features with an unmatched H-bond acceptor (Fig 3).

### Hypo-1 validation

**Fig 4:**
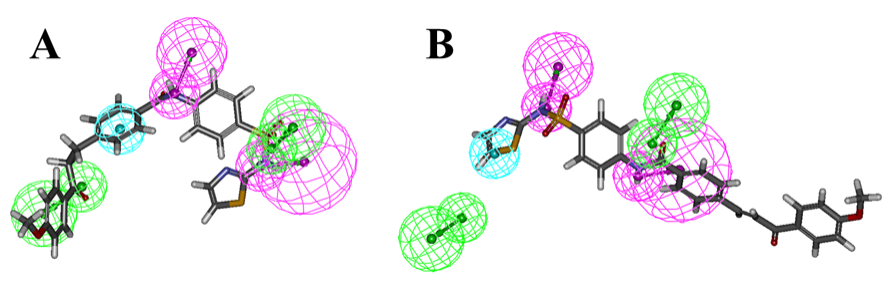
The active and less active compound from the test set aligned with Hypo-1.

The test set prediction was performed to validate the developed pharmacophore. The test set of 19 molecules which was classified into active, less active and inactive group, was screened with the final pharmacophore model. The resulting FitValues are shown in the Table 3. The FitValue > = 2.94 was observed to be a good threshold value which distinguished the active, less active and inactive molecules. In detail, 8 of 10 active, 5 of 5 less active and 2 of 3 inactive molecules were predicted correctly by the hypothesis. However, the distinct FitValue could not be obtained to distinguish between the less active/inactive molecules (Table 3). The hit rate of pharmacophore hypothesis suggested that the developed pharmacophore hypothesis can efficiently differentiate actives from less active and inactive molecules. Two active molecules were underestimated as less active/inactive, whereas one inactive molecule was overestimated as active by the hypothesis. Fig 4 shows the mapping of active and less active compound from the test set over the hypothesis. The highly active compound was mapped accurately to all the features while, in case of the less active molecules, one H-bond acceptor was missing and hydrophobic feature was mapped partially.

**Table 3:** FitValues and number of features matched for the test set molecules. FitValue of 2.94 was selected to distinguish active and less active molecules.

Sr. No.	Compound	FitValue	Feature matched	Activity Class
**1**	Cichoric acid	3.63	5	Active
**2**	Amentoflavone	3.55	5	Active
**3**	16	3.53	5	Active
**4**	11	3.48	5	Inactive
**5**	3	3.20	5	Active
**6**	4	3.21	5	Active
**7**	5	3.19	5	Active
**8**	Chlorogenic acid	3.00	5	Active
**9**	8	2.94	5	Active
**10**	2	2.82	5	less active
**11**	9	2.66	5	Inactive
**12**	6	2.24	5	less active
**13**	7	2.13	5	less active
**14**	10	1.96	5	Inactive
**15**	1	2.95	4	less active
**16**	12	3.70	4	Inactive
**17**	13	3.01	4	less active
**18**	14	3.06	4	Active
**19**	15	3.06	3	Active

Finally, DecoyFinder1.1 was employed to generate small database (D) containing 444 molecules, which includes 12 actives and 436 decoys. This database was used to validate the final pharmacophore hypothesis (Hypo-1), whether it is capable of distinguishing the actives from decoys or not. The hypothesis Hypo1 was used as a 3D structural query to perform screening of decoy database and then accuracy, precision, sensitivity, and specificity were calculated. The hits were selected based on the FitValue. Furthermore, for the analysis of results, the enrichment factor (E-value) and goodness of hit score (GH) were calculated using the following formulae:

$$\;\text{E}=\frac{\left(\text{TP}\times \text{D}\right)}{\left(\text{HT}\times \text{A}\right)}$$ (3)

GH=(TP/4HtA)(3A+HT)×(1−(((Ht−TP))/((D−A)))) (4)

Where D is the total number of molecules of the database, A is the total number of actives, Ht is the total number of molecules screened by a pharmacophore model and TP represent the total number of active molecules screened. PTP1B pharmacophore showed an E value of 26.42 (S1 Table). The calculated GH score was greater than 0.5 and it specifies that the quality of developed pharmacophore was significant (S1 Table).

From the overall validation results, we felt assured that the pharmacophore hypothesis could discriminate between the active and the less active molecules. Hence, we used this hypothesis for virtual screening to select or discriminate between suitable PTP1B allosteric inhibitors.

### Pharmacophore development of TCPTP inhibitors

The representative cluster conformations of Model-4, Model-5 and Model-6 were used for the generation of receptor based features. There were 1, 2 and 2 representative conformations for Model-4, Model-5 and Model-6, respectively. Each conformation was subjected to the identification of receptor based features in the LigandScout software. These five pharmacophore hypotheses are shown in S4 Fig.

**Fig 5:**
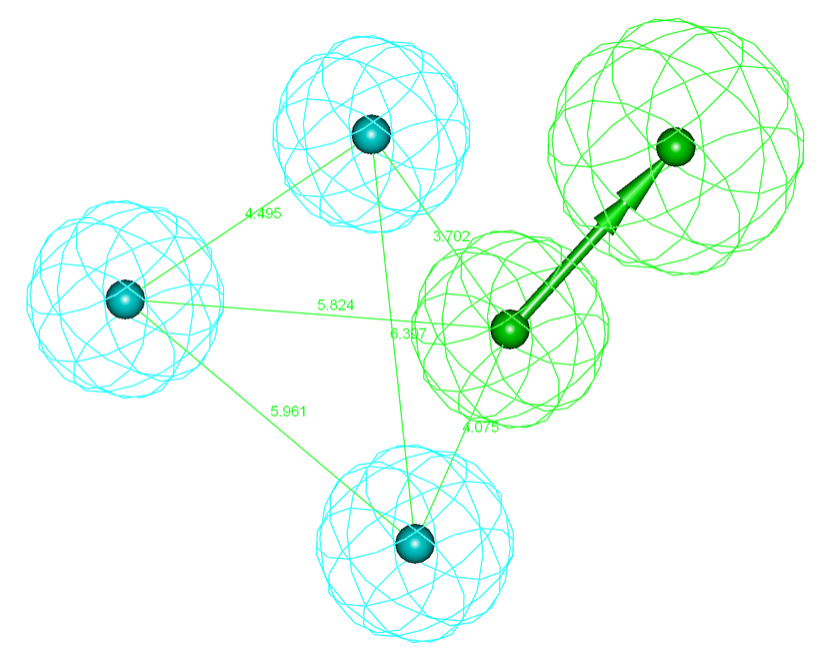
Receptor based pharmacophore model of TCPTP allosteric inhibitors generated by LigandScout. Pharmacophore features are: hydrogen-bond acceptor (green) and hydrophobic (cyan).

Pharmacophore model generated for the cluster-1 of TCPTP-Inhibitor-3 complex was used as the reference pharmacophore. All the four hypotheses were aligned to each other, features that are common were retained and a combined pharmacophore (Hypo-2) was generated. It contained the four features as shown in the Fig 5.

**Fig 6:**
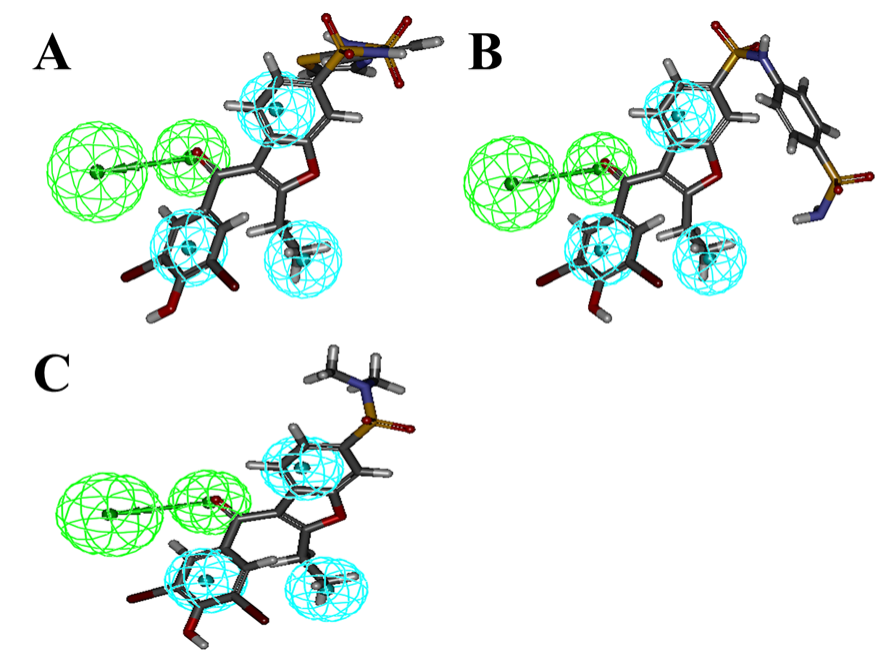
Pharmacophore mapping with training set molecules, Inhbitior-1 (A), Inhibitor-2 (B) and Inhibitor-3 matching all the features.

All features of model were nicely mapped with the corresponding chemical functional groups of Inhibitor 1, 2 and 3 in the training set (Fig 6).

FitValues 3.66, 3.69 and 3.67 was observed for them respectively. It was further tested with the known allosteric inhibitor, Molecule-16. It was also fitted to all the features with the FitValue of 3.82. However, this pharmacophore needs to be validated with more number of molecules. This FitValue of 3.66 was considered as the threshold value to identify the screening compounds during the virtual screening of database molecules.

### Virtual screening

The Specs databases, which comprised of 393932 compounds, were filtered using Lipinski’s rule of five. Further, the 265416 compounds obtained were screened through several toxicity filters, such as carcinogenicity, chromosome damage, genotoxicity, hERG channel inhibition, hepatotoxicity, mutagenicity and thyroid toxicity using DEREK. After applying toxicity filter 92276 compounds were obtained and subsequently screened by the validated PTP1B pharmacophore hypothesis. 21509 compounds were mapped to the pharmacophore hypothesis, which included some compounds structurally similar to that of existing inhibitors, and some novel scaffolds were also identified. Compound matching 4 features were also selected as they resemble the less active compounds and can identify novel scaffolds. A set of 656 compounds were obtained as hits after applying the filter criterion of FitValue > = 2.94. These compounds were then screened through the TCPTP pharmacophore and received a hit count of 243. Molecules showing FitValue > = 3.66 were believed to be TCPTP inhibitors. Removing these molecules, a set of selective 94 compounds were obtained. Out of these, 35 compounds having QED value > 0.50 were selected and subjected to further analysis by molecular docking to avoid false-positive hits from virtual screening. The 23 molecules showed proper orientation and the desired interactions in the active site of the PTP1B.

**Fig 7:**
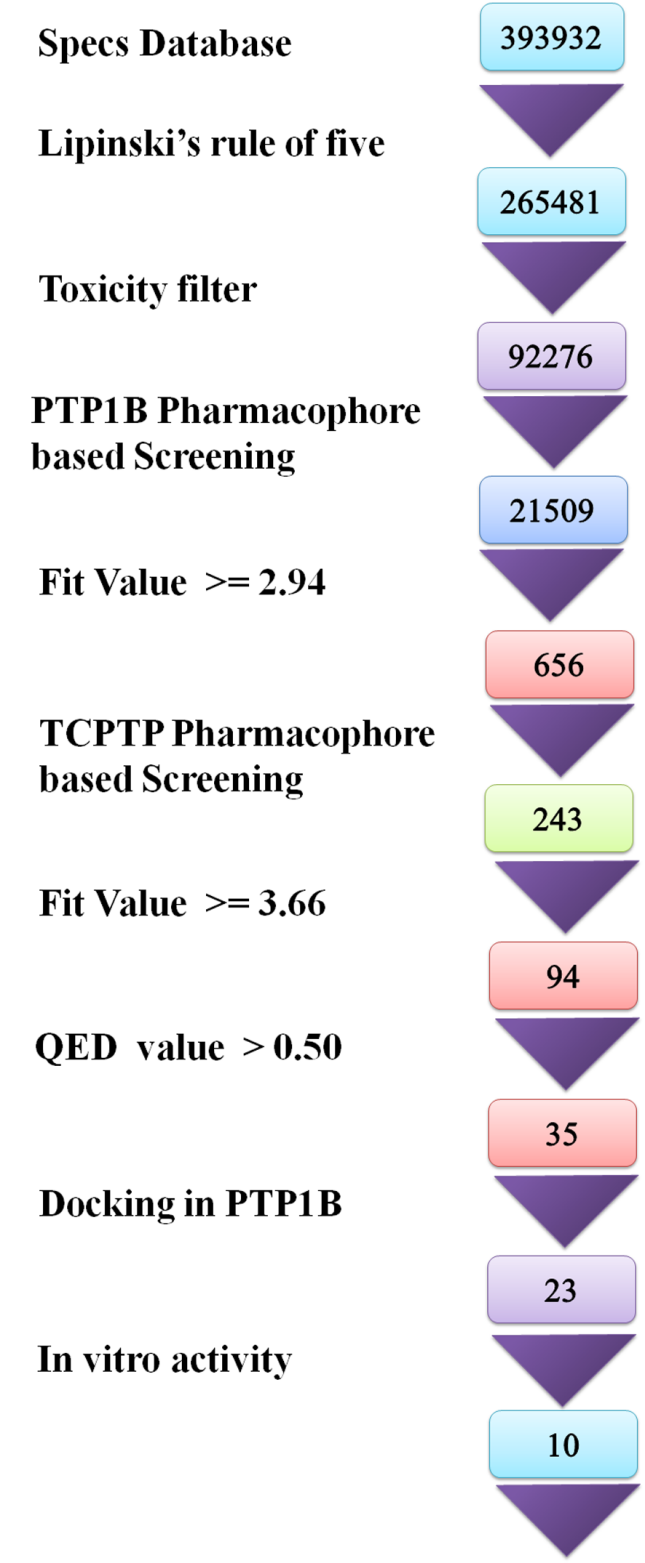
The sequential steps followed in the identification of novel allosteric inhibitors.

Sequential virtual screening was performed as depicted in the flowchart (Fig 7).

### Docking analysis

Crystal structures have shown H-bond interactions of inhibitors with the residues Asn193 and Glu276. These two interactions were observed to be consistent throughout the simulation. Hence, these interactions were considered to be essential during the selections of compounds that have matched with PTP1B pharmacophore. The compounds interacting with key residues viz., Leu192 and Phe196 of α3 helix, Phe280 and Glu276 of α6 helix, Trp291, Val287 and Gln288 from α7 helix, and Ser285 and Ser286 from α6-α7 loop were selected as potential hits. The docking scores of the 23 potential hits acquired after the virtual screening protocol are shown in Table 4.

**Table 4:** The glide score and FitValues for the of top 23 hits obtained from the virtual hits.

Sr. No	Name	Docking score	FitValue
**1**	NIPER-1	-9.65	4.01
**2**	NIPER-2	-9.85	3.76
**3**	NIPER-3	-8.70	3.65
**4**	NIPER-4	-9.45	3.56
**5**	NIPER-5	-8.47	3.55
**6**	NIPER-6	-9.52	3.46
**7**	NIPER-7	-9.23	3.45
**8**	NIPER-8	-8.59	3.42
**9**	NIPER-9	-8.83	3.39
**10**	NIPER-10	-8.92	3.39
**11**	NIPER-11	-8.67	3.37
**12**	NIPER-12	-8.82	3.35
**13**	NIPER-13	-9.23	3.35
**14**	NIPER-14	-8.87	3.25
**15**	NIPER-15	-9.16	3.24
**16**	NIPER-16	-8.71	3.23
**17**	NIPER-17	-9.47	3.21
**18**	NIPER-18	-8.73	3.20
**19**	NIPER-19	-8.92	3.16
**20**	NIPER-20	-9.01	3.15
**21**	NIPER-21	-9.13	2.99
**22**	NIPER-22	-9.15	2.99
**23**	NIPER-23	-9.20	2.96

**Fig 8:**
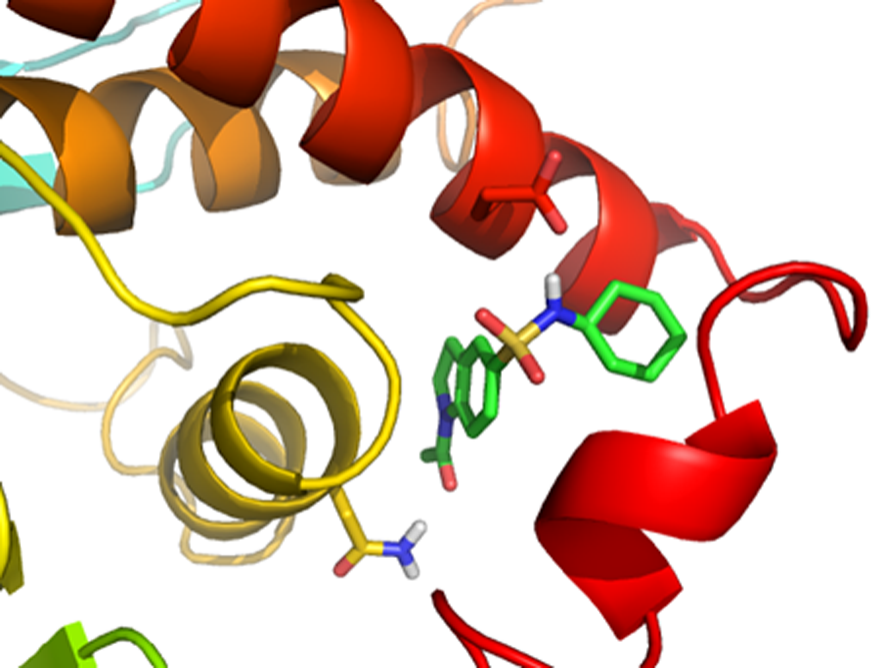
Interactions of molecule NIPER-23 at the allosteric site of PTP1B.

The docking scores and FitValue suggested that the identified hits have affinity towards PTP1B. They interacted mainly with the Phe280 and Trp291 by strong van der Waals interactions. Asn193 formed H-bond interactions through side chain amino nitrogen. Compounds with high docking scores spanned towards the α6 helix and α6-α7 loop residues making interactions with the Glu276, Gly283 and Asp284. They also made interactions with the Val287 of α7 helix. Docked pose of NIPER-23, showing interactions with these residues, is displayed in Fig 8.

### Geometrical criteria analysis

The conformations generated during the molecular dynamics of PTP1B-hit complexes were subjected to the geometric criteria analysis. Six criteria values for each conformation were calculated and averaged over the period of 20 ns production phase simulation. Their values are shown in the.

It can be inferred from the S2 Table that the complexes of hit molecule follow the criteria values of open state PTP1B structures. In addition, criterion that monitors the bending of WPD loop over phosphotyrosine was found to have low average values compared to the average values observed in the absence of these inhibitors. This can be attributed to the length of simulation period i.e. 25 ns. This demonstrates that the hit molecules can potentially act as allosteric inhibitors.

### Activity of compounds

**Fig 9:**
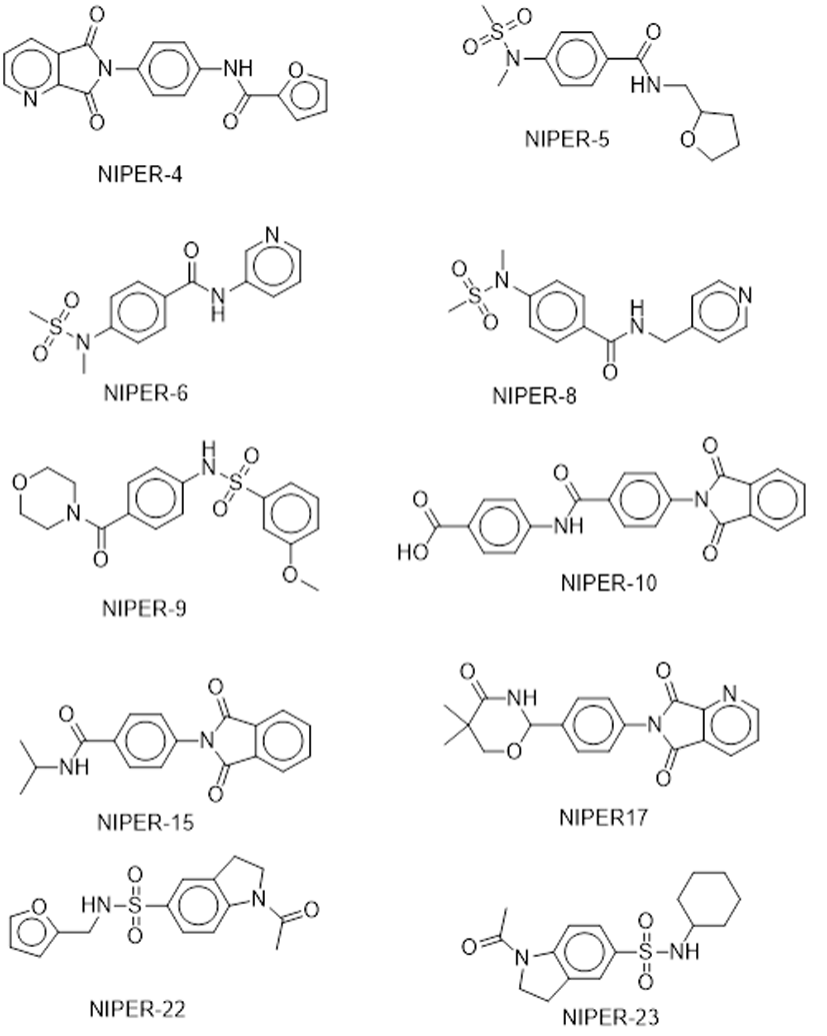
Structure of the molecules showing *in vitro* PTP1B inhibition.

These compounds were tested for in vitro PTP1B inhibition activity. Activity has been performed using p-nitrophenyl phosphate (pNPP) as the small molecule substrate and PTP1B of length 1–298. Percent inhibition of PTP1B was tested at concentration of 1.25 μM. Among the 23 tested, 10 compounds showed inhibitory activity against PTP1B. These compounds are shown in Fig 9 and their activity values are shown in Table 5. It suggest that molecules may be binding to the allosteric site. However, kinetic or mutational assays have not been performed to validate the binding site and allosteric mechanism of experimentally active molecules. In addition, selectivity of these inhibitors towards PTP1B needs to be established.

**Table 5:** Compounds that shows inhibition of PTP1B at 1.25 μM concentration in *in vitro* assays.

Sr. No.	1	2	3	4	5	6	7	8	9	10
Name	NIPER-4	NIPER-5	NIPER-6	NIPER-8	NIPER-9	NIPER-10	NIPER-15	NIPER-17	NIPER-22	NIPER-23
**1.25 μM**	37	40	42	45	47	56	55	10	44	28

### Permeability analysis

Different pharmacokinetic properties e.g. Caco-2 cell permeability, MDCK cell permeability and human intestinal absorption (HIA) were predicted for the potential hits (Table 6).

**Table 6:** Predicted human intestinal absorption (HIA) values for the active PTP1B inhibitors.

Name	Caco-permeability^a^ (nm/sec)	MDCK-permeability^b^ (nm/sec)	HIA^c^ (%)
**NIPER-4**	148.70	63.06	77.83
**NIPER-5**	1045.29	582.43	89.18
**NIPER-6**	950.21	525.36	88.78
**NIPER-8**	820.04	448.03	89.29
**NIPER-9**	580.65	315.82	88.73
**NIPER-10**	7.80	3.31	59.53
**NIPER-15**	500.22	246.33	77.36
**NIPER-17**	263.55	356.00	86.00
**NIPER-22**	582.32	476.57	54.00
**NIPER 23**	656.33	556.00	79.20

a, b—< 25 is poor, > 500 is great

c—< 25% is poor, > 80% is high

Caco-2 cells are a model for the gut-blood barrier non-active transport. MDCK cells are considered to be a good mimic for the non-active transport in blood-brain barrier. Human intestinal absorption correlates well with human oral absorption and the assessment uses a knowledge-based set of rules, including checking for suitable values of oral absorption, number of metabolites, number of rotatable bonds, logP, solubility and cell permeability. Except molecule NIPER-10 all molecules showed a high caco-2 permeability, MDCK permeability and HIA values, demonstrating that the allosteric inhibitors are potentially permeable compounds.

## Supporting information

S1 Fig Cartoon representation of PTP1B protein structural features. (TIF)

S2 Fig Structure of Inhibitor-1, Inhibitor-2 and Inhibitor-3 bound in the co-crystal structure 1T48, 1T49 and 1T4J, respectively. (TIF)

S3 Fig Modelling residues 278–293 and allosteric inhibitors in the TCPTP structure 1L8K. Figure shows sequence alignment of TCPTP and PTP1B structures. Structures 1T48, 2F6F and 1L8K used as template to build the TCPTP—Inhibitor-1 complex of 293 residues (Model-4). Procedure is repeated by replacing 1T48 by 1T49 to build TCPTP—Inhibitor-2 complex (Model-5) and by 1T4J to build TCPTP—Inhibitor-3 complex (Model-6).
(TIF)

S4 Fig 3D and 3D depiction pharmacophore features identified in the representative conformations of (A) Model-1, (B) Model-2, (C) Model-3, (D) Model-4, (E) Model-5 and (F) Model-6.
(PDF)

S1 Table The statistical parameters obtained from decoy dataset. (PDF)

S2 Table Seven geometric criteria values for the five model systems calculated for the conformations generated during production MD simulations. For each criterion average values are shown in Å. Direction of arrow indicates the higher (**↑**) or lower (↓) average value for the models containing allosteric inhibitor compared to the models-12 (PTP1B + pTyr).
(PDF)

## References

[1] Alonso A, Sasin J, Bottini N, Friedberg I, Friedberg I, Osterman A, et al (2004) Protein tyrosine phosphatases in the human genome. Cell 117: 699–711. doi: 10.1016/j.cell.2004.05.018 15186772 10.1016/j.cell.2004.05.018

[2] Barford D (1996) Molecular mechanisms of the protein serine/threonine phosphatases. Trends Biochem Sci 21: 407–412. 8987393 10.1016/s0968-0004(96)10060-8

[3] Hendriks WJ, Elson A, Harroch S, Pulido R, Stoker A, den Hertog J (2013) Protein tyrosine phosphatases in health and disease. FEBS J 280: 708–730. doi: 10.1111/febs.12000 22938156 10.1111/febs.12000

[4] Tonks NK (2013) Protein tyrosine phosphatases—from housekeeping enzymes to master regulators of signal transduction. FEBS J 280: 346–378. doi: 10.1111/febs.12077 23176256 10.1111/febs.12077 PMC3662559

[5] Barford D, Jia Z, Tonks NK (1995) Protein tyrosine phosphatases take off. Nat Struct Biol 2: 1043–1053. 8846213 10.1038/nsb1295-1043

[6] Barford D, Flint AJ, Tonks NK (1994) Crystal structure of human protein tyrosine phosphatase 1B. Science 263: 1397–1404. 8128219

[7] Frangioni JV, Beahm PH, Shifrin V, Jost CA, Neel BG The nontransmembrane tyrosine phosphatase PTP-1B localizes to the endoplasmic reticulum via its 35 amino acid C-terminal sequence. Cell 68: 545–560. 1739967 10.1016/0092-8674(92)90190-n

[8] Dube N, Tremblay ML (2005) Involvement of the small protein tyrosine phosphatases TC-PTP and PTP1B in signal transduction and diseases: from diabetes, obesity to cell cycle, and cancer. Biochim Biophys Acta 1754: 108–117. doi: 10.1016/j.bbapap.2005.07.030 16198645 10.1016/j.bbapap.2005.07.030

[9] Elchebly M, Payette P, Michaliszyn E, Cromlish W, Collins S, Loy AL, et al (1999) Increased insulin sensitivity and obesity resistance in mice lacking the protein tyrosine phosphatase-1B gene. Science 283: 1544–1548. 10066179 10.1126/science.283.5407.1544

[10] Klaman LD, Boss O, Peroni OD, Kim JK, Martino JL, Zabolotny JM, et al (2000) Increased energy expenditure, decreased adiposity, and tissue-specific insulin sensitivity in protein-tyrosine phosphatase 1B-deficient mice. Mol Cell Biol 20: 5479–5489. 10891488 10.1128/mcb.20.15.5479-5489.2000 PMC85999

[11] Zabolotny JM, Bence-Hanulec KK, Stricker-Krongrad A, Haj F, Wang Y, Minokoshi Y, et al (2002) PTP1B regulates leptin signal transduction in vivo. Dev Cell 2: 489–495. 11970898 10.1016/s1534-5807(02)00148-x

[12] Liu F, Hill DE, Chernoff J (1996) Direct binding of the proline-rich region of protein tyrosine phosphatase 1B to the Src homology 3 domain of p130(Cas). J Biol Chem 271: 31290–31295. 8940134 10.1074/jbc.271.49.31290

[13] Frangioni JV, Beahm PH, Shifrin V, Jost CA, Neel BG (1992) The nontransmembrane tyrosine phosphatase PTP-1B localizes to the endoplasmic reticulum via its 35 amino acid C-terminal sequence. Cell 68: 545–560. 1739967 10.1016/0092-8674(92)90190-n

[14] Jia Z, Barford D, Flint AJ, Tonks NK (1995) Structural basis for phosphotyrosine peptide recognition by protein tyrosine phosphatase 1B. Science 268: 1754–1758. 7540771 10.1126/science.7540771

[15] Kamerlin SC, Rucker R, Boresch S (2006) A targeted molecular dynamics study of WPD loop movement in PTP1B. Biochem Biophys Res Commun 345: 1161–1166. doi: 10.1016/j.bbrc.2006.04.181 16713994 10.1016/j.bbrc.2006.04.181

[16] Kamerlin SC, Rucker R, Boresch S (2007) A molecular dynamics study of WPD-loop flexibility in PTP1B. Biochem Biophys Res Commun 356: 1011–1016. doi: 10.1016/j.bbrc.2007.03.093 17408595 10.1016/j.bbrc.2007.03.093

[17] Peters GH, Frimurer TM, Andersen JN, Olsen OH (1999) Molecular dynamics simulations of protein-tyrosine phosphatase 1B. I. ligand-induced changes in the protein motions. Biophys J 77: 505–515. doi: 10.1016/S0006-3495(99)76907-9 10388775 10.1016/S0006-3495(99)76907-9 PMC1300347

[18] Peters GH, Frimurer TM, Andersen JN, Olsen OH (2000) Molecular dynamics simulations of protein-tyrosine phosphatase 1B. II. substrate-enzyme interactions and dynamics. Biophys J 78: 2191–2200. doi: 10.1016/S0006-3495(00)76768-3 10777720 10.1016/S0006-3495(00)76768-3 PMC1300813

[19] Byon JCH, Kusari AB, Kusari J (1998) Protein-tyrosine phosphatase-1B acts as a negative regulator of insulin signal transduction. Molecular and cellular biochemistry 182: 101–108. 9609119

[20] Kenner KA, Anyanwu E, Olefsky JM, Kusari J (1996) Protein-tyrosine phosphatase 1B is a negative regulator of insulin-and insulin-like growth factor-I-stimulated signaling. Journal of Biological Chemistry 271: 19810–19816. 8702689 10.1074/jbc.271.33.19810

[21] Iversen LF, Moller KB, Pedersen AK, Peters GH, Petersen AS, Andersen HS, et al (2002) Structure determination of T cell protein-tyrosine phosphatase. J Biol Chem 277: 19982–19990. doi: 10.1074/jbc.M200567200 11907034 10.1074/jbc.M200567200

[22] You-Ten KE, Muise ES, Itie A, Michaliszyn E, Wagner J, Jothy S, et al (1997) Impaired bone marrow microenvironment and immune function in T cell protein tyrosine phosphatase-deficient mice. J Exp Med 186: 683–693. 9271584 10.1084/jem.186.5.683 PMC2199020

[23] van Montfort RL, Congreve M, Tisi D, Carr R, Jhoti H (2003) Oxidation state of the active-site cysteine in protein tyrosine phosphatase 1B. Nature 423: 773–777. doi: 10.1038/nature01681 12802339 10.1038/nature01681

[24] Brandao TA, Hengge AC, Johnson SJ (2010) Insights into the reaction of protein-tyrosine phosphatase 1B: crystal structures for transition state analogs of both catalytic steps. J Biol Chem 285: 15874–15883. doi: 10.1074/jbc.M109.066951 20236928 10.1074/jbc.M109.066951 PMC2871455

[25] Wiesmann C, Barr KJ, Kung J, Zhu J, Erlanson DA, Shen W, et al (2004) Allosteric inhibition of protein tyrosine phosphatase 1B. Nature structural & molecular biology 11: 730–737. 10.1038/nsmb803 15258570

[26] Barr K, Fahr B, Hansen S, Wiesmann C (2004) Compounds that modulate the activity of PTP-1B and TC-PTP. US.

[27] Shinde RN, Sobhia ME (2013) Binding and discerning interactions of PTP1B allosteric inhibitors: Novel insights from molecular dynamics simulations. Journal of Molecular Graphics and Modelling 45: 98–110. doi: 10.1016/j.jmgm.2013.08.001 24012873 10.1016/j.jmgm.2013.08.001

[28] Montalibet J, Skorey K, McKay D, Scapin G, Asante-Appiah E, Kennedy BP (2006) Residues distant from the active site influence protein-tyrosine phosphatase 1B inhibitor binding. J Biol Chem 281: 5258–5266. doi: 10.1074/jbc.M511546200 16332678 10.1074/jbc.M511546200

[29] Li S, Zhang J, Lu S, Huang W, Geng L, Shen Q, et al (2014) The Mechanism of Allosteric Inhibition of Protein Tyrosine Phosphatase 1B. PLoS ONE 9: e97668 doi: 10.1371/journal.pone.0097668 24831294 10.1371/journal.pone.0097668 PMC4022711

[30] Bharatham K, Bharatham N, Kwon YJ, Lee KW (2008) Molecular dynamics simulation study of PTP1B with allosteric inhibitor and its application in receptor based pharmacophore modeling. J Comput Aided Mol Des 22: 925–933. doi: 10.1007/s10822-008-9229-0 18685809 10.1007/s10822-008-9229-0

[31] Olmez EO, Alakent B (2011) Alpha7 helix plays an important role in the conformational stability of PTP1B. J Biomol Struct Dyn 28: 675–693. doi: 10.1080/07391102.2011.10508599 21294582 10.1080/07391102.2011.10508599

[32] Baskaran SK, Goswami N, Selvaraj S, Muthusamy VS, Lakshmi BS (2012) Molecular dynamics approach to probe the allosteric inhibition of PTP1B by chlorogenic and cichoric acid. J Chem Inf Model 52: 2004–2012. doi: 10.1021/ci200581g 22747429 10.1021/ci200581g

[33] Lee JY, Jung KW, Woo ER, Kim Y (2008) Docking study of biflavonoids, allosteric inhibitors of protein tyrosine phosphatase 1B. Bulletin of the Korean Chemical Society 29: 1479–1484.

[34] Cui W, Cheng YH, Geng LL, Liang DS, Hou TJ, Ji MJ (2013) Unraveling the Allosteric Inhibition Mechanism of PTP1B by Free Energy Calculation Based on Umbrella Sampling. J Chem Inf Model 53: 1157–1167. doi: 10.1021/ci300526u 23621621 10.1021/ci300526u

[35] Eswar N, Webb B, Marti-Renom MA, Madhusudhan MS, Eramian D, Shen M, et al (2007) Comparative protein structure modeling using Modeller. Curr Protoc Protein Sci: 2.9. 1–2.9. 31. 10.1002/0471140864.ps0209s50 18429317

[36] Nan F, Li J, Wei Y, Zhang W, Li J, Shi L (2008) Protein tyrosine phosphatase 1b inhibitor, preparation methods and uses thereof. In: Shanghai Institute Of Materia Medica CAOFSNZRZH-TPPNDS, editor. WO.

[37] Hess B, Kutzner C, van der Spoel D, Lindahl E (2008) GROMACS 4: algorithms for highly efficient, load-balanced, and scalable molecular simulation. Journal of Chemical Theory and Computation 4: 435–447. doi: 10.1021/ct700301q 26620784 10.1021/ct700301q

[38] Daura X, Gademann K, Jaun B, Seebach D, van Gunsteren WF, Mark AE (1999) Peptide folding: when simulation meets experiment. Angew Chem Int Ed 38: 236–240.

[39] Ehrlich P (1909) Über den jetzigen Stand der Chemotherapie. Berichte der deutschen chemischen Gesellschaft 42: 17–47.

[40] Wermuth C-G, Ganellin CR, Lindberg P, Mitscher LA (1998) Chapter 36. Glossary of Terms Used in Medicinal Chemistry (IUPAC Recommendations 1997 In: James AB, editor. Annual Reports in Medicinal Chemistry: Academic Press pp. 385–395.

[41] Barnum D, Greene J, Smellie A, Sprague P (1996) Identification of common functional configurations among molecules. J Chem Inf Comput Sci 36: 563–571. 8690757 10.1021/ci950273r

[42] Li H, Sutter J, Hoffman R (2000) HypoGen: An Automated System for Generating 3D Predictive Pharmacophore Models In: Guner O, editor. Pharmacophore Perception, Development and Use in Drug Design: International University Line.

[43] Richmond NJ, Abrams CA, Wolohan PR, Abrahamian E, Willett P, Clark RD (2006) GALAHAD: 1. pharmacophore identification by hypermolecular alignment of ligands in 3D. J Comput Aided Mol Des 20: 567–587. doi: 10.1007/s10822-006-9082-y 17051338 10.1007/s10822-006-9082-y

[44] Dixon SL, Smondyrev AM, Knoll EH, Rao SN, Shaw DE, Friesner RA (2006) PHASE: a new engine for pharmacophore perception, 3D QSAR model development, and 3D database screening: 1. Methodology and preliminary results. J Comput Aided Mol Des 20: 647–671. doi: 10.1007/s10822-006-9087-6 17124629 10.1007/s10822-006-9087-6

[45] Wolber G, Langer T (2005) LigandScout: 3-D pharmacophores derived from protein-bound ligands and their use as virtual screening filters. J Chem Inf Model 45: 160–169. doi: 10.1021/ci049885e 15667141 10.1021/ci049885e

[46] Chen J, Lai L (2006) Pocket v.2: further developments on receptor-based pharmacophore modeling. J Chem Inf Model 46: 2684–2691. doi: 10.1021/ci600246s 17125208 10.1021/ci600246s

[47] Ortuso F, Langer T, Alcaro S (2006) GBPM: GRID-based pharmacophore model: concept and application studies to protein-protein recognition. Bioinformatics 22: 1449–1455. doi: 10.1093/bioinformatics/btl115 16567363 10.1093/bioinformatics/btl115

[48] Tang YB, Lu D, Chen Z, Hu C, Yang Y, Tian JY, et al (2013) Design, synthesis and insulin-sensitising effects of novel PTP1B inhibitors. Bioorg Med Chem Lett 23: 2313–2318. doi: 10.1016/j.bmcl.2013.02.073 23499238 10.1016/j.bmcl.2013.02.073

[49] Cereto-Massague A, Guasch L, Valls C, Mulero M, Pujadas G, Garcia-Vallve S (2012) DecoyFinder: an easy-to-use python GUI application for building target-specific decoy sets. Bioinformatics 28: 1661–1662. doi: 10.1093/bioinformatics/bts249 22539671 10.1093/bioinformatics/bts249

[50] Singh U, Gangwal RP, Prajapati R, Dhoke GV, Sangamwar AT (2012) 3D QSAR pharmacophore-based virtual screening and molecular docking studies to identify novel matrix metalloproteinase 12 inhibitors. Molecular Simulation 39: 385–396.

[51] Bickerton GR, Paolini GV, Besnard J, Muresan S, Hopkins AL (2012) Quantifying the chemical beauty of drugs. Nat Chem 4: 90–98. doi: 10.1038/nchem.1243 22270643 10.1038/nchem.1243 PMC3524573

[52] Cornell WD, Cieplak P, Bayly CI, Gould IR, Merz KM, Ferguson DM, et al (1995) A second generation force field for the simulation of proteins, nucleic acids, and organic molecules. Journal of the American Chemical Society 117: 5179–5197.

