# Supplementary figures and images for: Screening and identification of potential PTP1B allosteric inhibitors using in silico and in vitro approaches
Source: PLoS One. 2018 Jun 18;13(6):e0199020. doi: 10.1371/journal.pone.0199020 (PMC6005499; doi:10.1371/journal.pone.0199020)

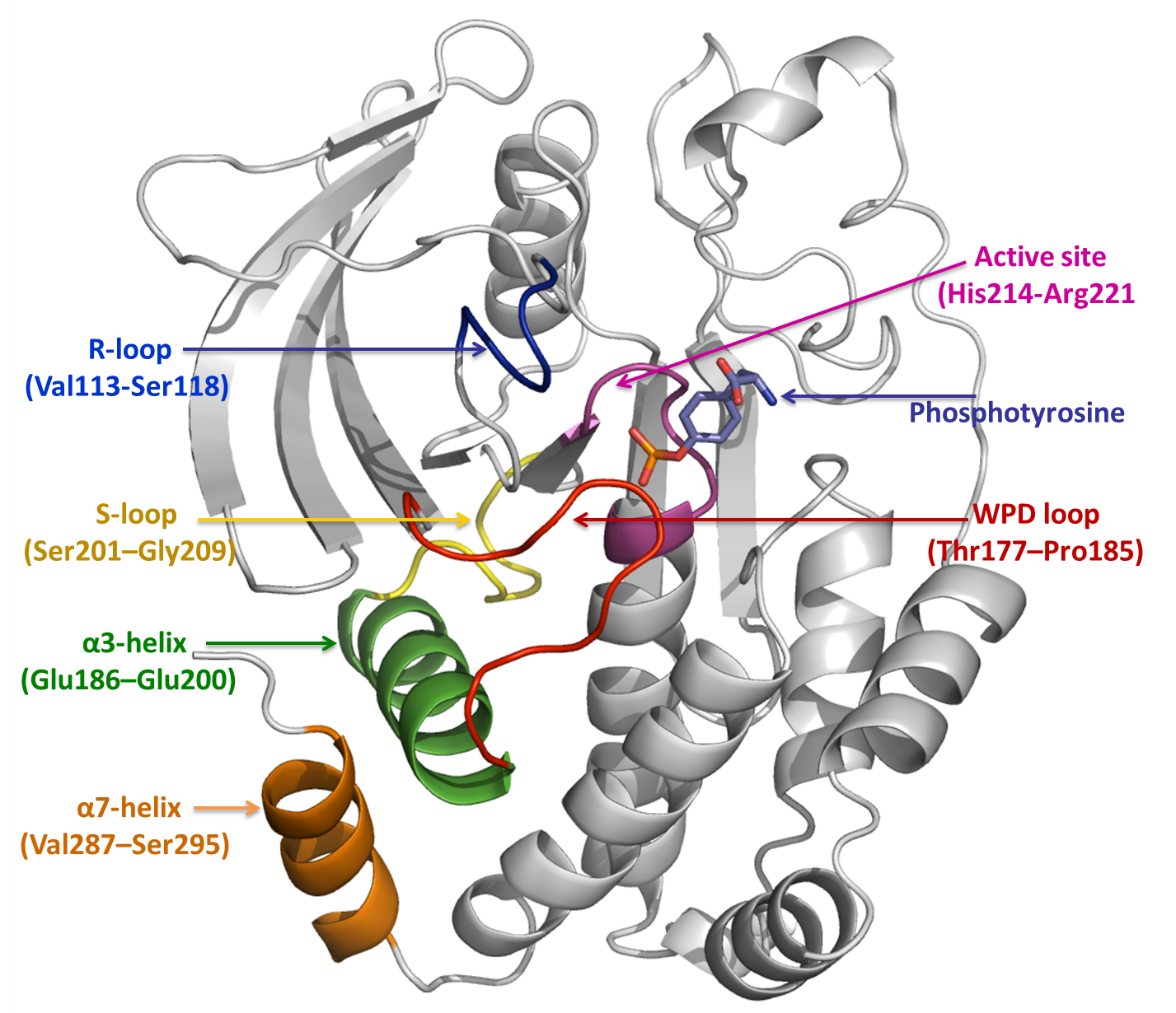

Supplement: S1 Fig — (TIF) [file pone.0199020.s001.tif]

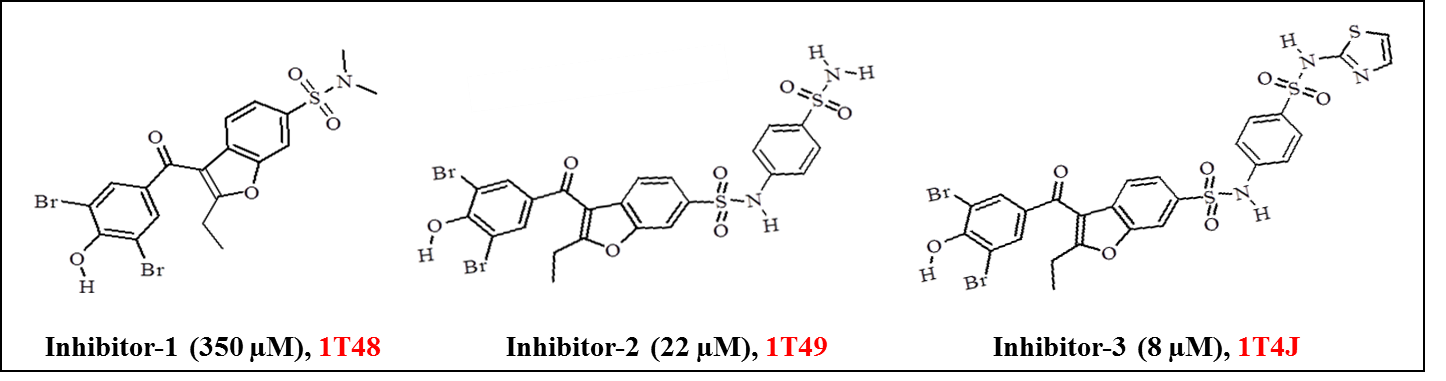

Supplement: S2 Fig — (TIF) [file pone.0199020.s002.tif]

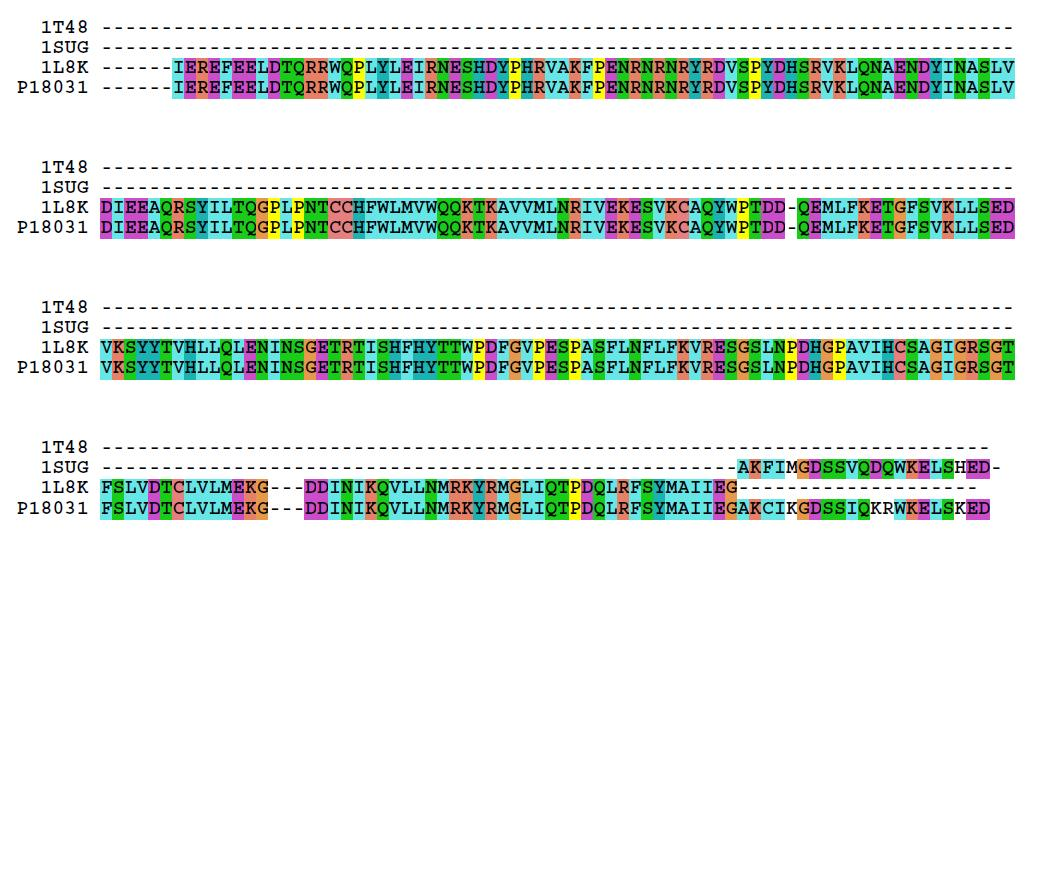

Supplement: S3 Fig — Figure shows sequence alignment of TCPTP and PTP1B structures. Structures 1T48, 2F6F and 1L8K used as template to build the TCPTP—Inhibitor-1 complex of 293 residues (Model-4). Procedure is repeated by replacing 1T48 by 1T49 to build TCPTP—Inhibitor-2 complex (Model-5) and by 1T4J to build TCPTP—Inhibitor-3 complex (Model-6). (TIF) [file pone.0199020.s003.tif]
