# Supplementary material for: Screening and identification of potential PTP1B allosteric inhibitors using in silico and in vitro approaches
Source: PLoS One. 2018 Jun 18;13(6):e0199020. doi: 10.1371/journal.pone.0199020 (PMC6005499; doi:10.1371/journal.pone.0199020)
Supplement: S4 Fig — 3D and 3D depiction pharmacophore features identified in the representative conformations of (A) Model-1, (B) Model-2, (C) Model-3, (D) Model-4, (E) Model-5 and (F) Model-6. (PDF) [file pone.0199020.s004.pdf]

		Cartoon representation of representative conformation	Protein-ligand Interactions
Model-1	Cluster-1	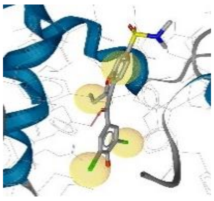	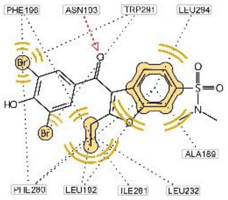
	Cluster-2	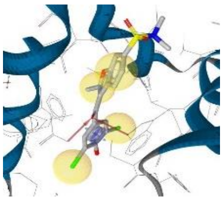	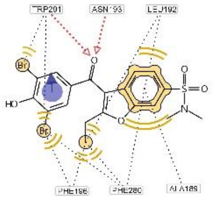
	Cluster-3	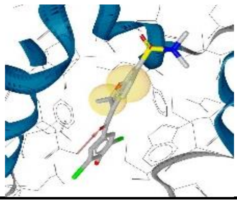	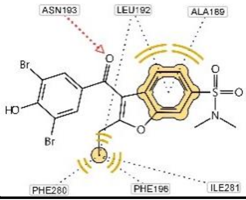
Model-2	Cluster-1	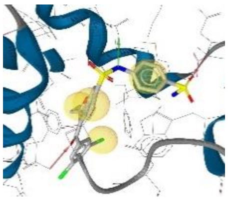	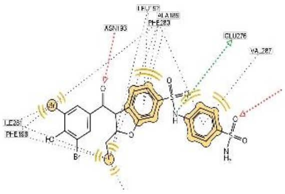
	Cluster-2	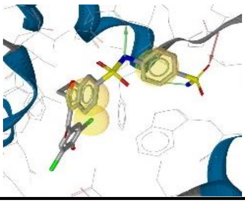	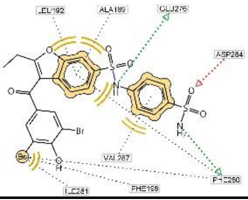
Model-3	Cluster-1	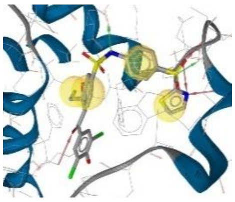	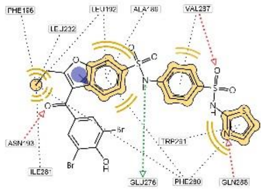
	Cluster-2	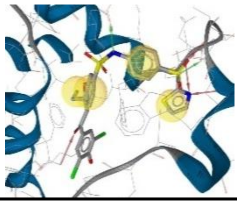	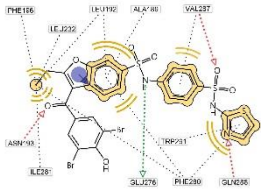
Model-4	Cluster-1	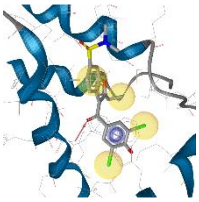	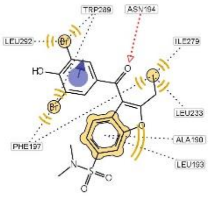

Model-5	Cluster-1	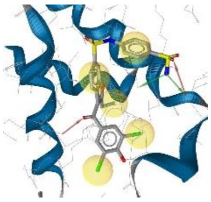	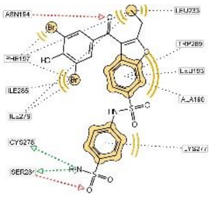
	Cluster-2	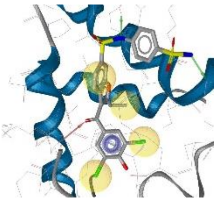	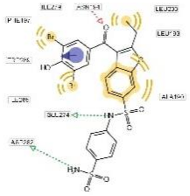
Model-6	Cluster-1	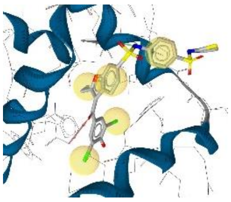	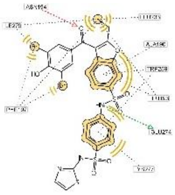
	Cluster-2	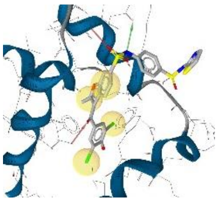	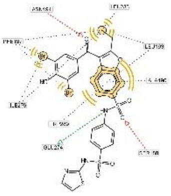

S4 Fig. 3D and 2D depiction pharmacophore features identified in the representative conformations of (A) Model-1, (B) Model-2, (C) Model-3, (D) Model-4, (E) Model-5 and (F) Model-6.
