# Supplementary material for: Screening and identification of potential PTP1B allosteric inhibitors using in silico and in vitro approaches
Source: PLoS One. 2018 Jun 18;13(6):e0199020. doi: 10.1371/journal.pone.0199020 (PMC6005499; doi:10.1371/journal.pone.0199020)
Supplement: S1 Table — (PDF) [file pone.0199020.s005.pdf]

S1 Table. The statistical parameters obtained from decoy dataset.

Sr. No.	Parameter	PTP1B pharmacophore
**1**	Total number of molecules in database (D)	444
**2**	Total Number of actives in database (A)	12
**3**	Total number of hit molecules from the database (Ht)	14
**4**	Total number of active molecules in hit list (Ha)	10
**5**	% Yield of actives [(Ha/Ht) × 100]	71.42
**6**	% Ratio of actives [(Ha/A) × 100]	83.33
**7**	True positive (TP)	10
**8**	True negative (TN)	428
**9**	False positives (FP = Ht-Ha)	14 - 10 = 4
**10**	False negatives (FN = A-Ha)	12 - 10 = 2
**11**	Accuracy = (TP+TN)/(TP+TN+FP+FN)	(10+428)/(10+428+4+2) = 0.98
**12**	Precision = TP/(TP+FP)	10/10+4 = 0.71
**13**	Sensitivity = TP/(TP+FN)	10/10+2 = 0.83
**14**	Specificity = TN/(TN+FP)	428/(428+4) = 0.99
**15**	Enrichment factor or enhancement (E)[b]	26.42
**16**	GH score (goodness of hit list)[a]	0.74

[a] - [((TP / 4HtA) / (3A + Ht)) × (1 - ((Ht - TP) / (D - A)))].
[b] - (TP × D) / (Ht × A)
