# Supplementary material for: Screening and identification of potential PTP1B allosteric inhibitors using in silico and in vitro approaches
Source: PLoS One. 2018 Jun 18;13(6):e0199020. doi: 10.1371/journal.pone.0199020 (PMC6005499; doi:10.1371/journal.pone.0199020)
Supplement: S2 Table — For each criterion average values are shown in Å. Direction of arrow indicates the higher (↑) or lower (↓) average value for the models containing allosteric inhibitor compared to the models-12 (PTP1B + pTyr). (PDF) [file pone.0199020.s006.pdf]

S2 Table. Seven geometric criteria values for the five model systems calculated for the conformations generated during production MD simulations. For each criterion average values are shown in Å. Direction of arrow indicates the higher (↑) or lower (↓) average value for the models containing allosteric inhibitor compared to the models-12 (PTP1B + pTyr).

	**Distance (Å)**						
**Criteria Number**	1	2	3	4	5	6	7
**Criteria**	Gly183(O) – Gln266(N_{ε})	Pro180(O) – Gly183(N)	Trp179(N_{ε}) – Gly183(O)	Ser187(O) – Phe191(N)	Ser187(O_{γ}) – Ser190(N)	Phe182(C_{α}) – Gly218(C_{α})	Trp179(O) – Arg221(N_{η})
**Model-12 (PTP1B + pTyr)**	6.08	4.75	3.27	5.08	5.17	16.24	5.14
**NIPER-2 complex**	6.12	4.80	3.15	5.16	5.20	16.32 ↑	5.16
**NIPER-7 complex**	6.15	4.88	3.36	5.12	5.12	16.35 ↑	5.24
**NIPER-8 complex**	6.00	4.82	3.31	5.10	5.15	16.35 ↑	5.09
**NIPER-9 complex**	6.04	4.77	3.20	5.00	5.25	16.30 ↑	5.12
